# Healing Effects of Luteolin Versus Silver Sulphadiazine on Second-Degree Burn Wounds in Animal Model

**DOI:** 10.31661/gmj.v13i.3351

**Published:** 2024-08-02

**Authors:** Seyed Alireza Salimi Tabatabaee, Fatemeh Karamali, Ghobad Rahimi, Seyed Abbas Mirmalek, Shima Shafagh, Hossein Sadeghi Hassan Abadi

**Affiliations:** ^1^ Trauma Research Center, Kashan University of Medical Sciences, Kashan, Iran; ^2^ Department of Cardiovascular Medicine, Kashan University of Medical Sciences, Kashan, Iran; ^3^ Department of Internal Medicine, North Khorasan University of Medical Sciences, Bojnurd, Iran; ^4^ Department of Surgery, Tehran Medical Sciences, Islamic Azad University, Tehran, Iran

**Keywords:** Luteolin, Burn Wound, Healing, Neutral Product, Silver Sulphadiazine

## Abstract

Background: Burn wounds are one of the most important injuries with significant mortality and morbidity. Although some treatments like silver sulphadiazine (SSD) ointment were introduced, the more effective as well as lower complications agents are under investigation. Current evidence shows that luteolin, as a flavonoid component of some fruits and vegetables, has potent anti-inflammatory and antioxidative properties. Hence this study aimed to investigate the healing effects of luteolin ointments on second-degree burn wounds in rat models and compared it with SSD. Materials and Methods: Thirty male rats were randomly divided into five equal groups. A 2×2 cm2 circled second-degree wound was induced on the dorsal surface of the rat neck. In the control group, rats received no any treatments while in the vehicle group rats received ointment base, i.e., eucerin. Rats in positive control and experimental groups were treated with SSD and luteolin ointments (i.e., contained 2 and 5 percent of luteolin), respectively. The treatments of rats were performed daily for 17 days and wound closer rate (WCR) was measured. Also, histopathological examinations graded the severity of tissue damages using the determination of collagen formation, re-epithelialization, angiogenesis rate, and polymorphological leukocyte density. Results: On the 17th day, WCR in control and vehicle groups was markedly lower than in both treatment and experimental groups (P0.005). Also, WCRs in the L2% and L5% groups were higher than the SSD group. Histopathological studies indicated more significant wound healing effects of L2% and L5% ointments versus SSD and eucerin treatments in terms of tissue-enhanced damage severity in a dose-dependent manner. Also, collagen formation and re-epithelialization were markedly more observed in the rats, which received luteolin ointments than in other groups. Conclusion: Our study revealed that based on WCR and histopathological examination findings, luteolin ointments could significantly enhance wound healing more than SSD (as the standard treatment) in the second-degree wounds rat model.

## Introduction

Burn injuries remain a significant public health concern worldwide, leading to high morbidity and mortality rates, as well as long-lasting physical and psychological effects on survivors [1][2]. Among the various types of burns, second-degree wounds are particularly challenging to treat due to their propensity for delayed healing, risk of infection, and potential for scar formation [3]. Current treatment options for second-degree burns often involve the use of topical agents to promote wound healing and reduce complications [4].

Silver sulfadiazine (SSD), a commonly used topical antimicrobial agent, has been a cornerstone of burn wound treatment for decades, owing to its broad-spectrum antimicrobial activity and ability to reduce infection rates [5]. However, concerns regarding its cytotoxic effects, delayed wound healing, and potential for microbial resistance have prompted the exploration of alternative therapeutic approaches [6].

Luteolin, a natural flavonoid compound found in certain fruits and vegetables, has some important potential therapeutic properties, including anti-inflammatory [7], antioxidant [8], and wound-healing effects [9].

Previous studies have suggested that luteolin may accelerate the healing process of various types of wounds, including burns, by modulating inflammatory responses, promoting tissue regeneration, and enhancing collagen synthesis [10][11]. However, the specific effects of luteolin ointment on second-degree burn wounds have not been comprehensively investigated.

Hence, this study aimed to investigate the healing effects of topical luteolin ointments compared to SSD on second-degree burn wounds in rats.

## Materials and Methods

**Table:** Table1. The grading scoring system of evaluation of tissue damage of burned wound [13]

Parameters	Collagen formation	Polymorphonuclear leukocytes	Degree of angiogenesis	Re-epithelialization
Grade 0	None	None	None	None
Grade 1	Low	Low	Less than five veins	Partial
Grade 2	Moderate	Moderate	6-10 veins	Complete but immature
Grade 3	High	High	More than 10 veins	Complete but mature

Preparation Treatment Ointments

In this study, eucerin was applied as the basement of used ointments. Briefly, for preparing Luteolin ointments 2% (w/w) and 5%, the 2 and 5 g of luteolin were combined with 98 and 95 g of eucerin, respectively. Then, all the ointment was stored in the proper place till the end of the study. All the ointments were applied topically once a day for 17 consecutive days.

Induction of Second-degree Burn Wound

Based on Abbasy et al. [4], an intraperitoneal injection of 100/10 mg ketamine/xylazine was used to anesthetize the rats. On the dorsal part of the animals’ necks, the skin was shaved and a 2×2 cm2 full-thickness circular second-degree burn wound was performed using electrical heaters with 110°C heat for 10 seconds.

Animals and Groups

Thirty male Wistar rats, weighing 200-250 g, were housed individually in cages with a 12-hour light-dark cycle at 23°C, and free access to pellet diet and water ad libitum for a week prior to experiments.

All the rats were randomly divided into five groups (n=6 per group) as follows:

-Negative control group (NC): Rats were not received any treatments

-Vehicle group: To eliminate the possible effect of the base of the ointments, rats in this group received only eucerin ointment

-Positive control group: Rats in this group received SSD 1% ointment as standard treatment

-Experimental groups: Rats in L2% and L5% groups were treated with luteolin ointments 2 and 5 percent, respectively

Wound Healing Measurement

Wound healing was assessed by calculated wound closure rate (WCR), and monitored the time needed for its complete closure. The wound surface area was measured every 24 hours from the creation of the wound (day zero) until its complete healing and closure. The animal was placed in a crouching position and the wound outline was carefully drawn on a transparent plastic sheet using a fine-tip color-stable marker.

Then, the wound surface area was precisely calculated using Image J software, and the percentage of WCR was calculated for different days according to the following formula [12]:

WCR (%) = (wound area at day one) - (wound area at day X)/ (wound area at day one) ×100

Samples Collection and Histological Analysis

All rats were sacrificed with an overdose of pentobarbital at the end of the last days of treatment. Then, biopsy specimens were obtained from wound site tissue. To assess histological changes, samples were stored in 10% buffered formalin. Each tissue specimen was sectioned into a set of 3-4 mm thick sections. Hematoxylin and Eosin (H&E) were used to stain the tissue, and microscopic photographs were taken at proper magnifications.

Histopathologic findings including collagen formation, inflammation, neovascularization, and re-epithelialization were recorded for each group. Wound healing was evaluated using a grading system introduced by Lukiswanto et al. [13]. Regarding Table-1, all the wounds were classified into four grades in which grade 0 is showed worsted and grade 3 is the best healing response.

Ethical Consideration

All procedures were performed according to the Guide for the Care and Use of Laboratory Animals (NIH publication number 86-23, 1985 edition). Also, this study was approved by the Research Ethics Committees of Laboratory Animals of Kashan University of Medical Sciences and Health Services (code: IR.KAUMS.AEC.1402.014).

Statistical Analysis

All data were expressed as mean ± standard deviations (SD) and were analyzed using GraphPad Prism software (version 6.01, GraphPad, La Jolla, CA, USA). Also, one-way analysis of variance (ANOVA) followed by Tukey’s multiple comparison tests, as well as, the Mann-Whitney test for nonparametric data were applied. A P=0.05 was considered as significance level.

## Results

**Figure-1:**
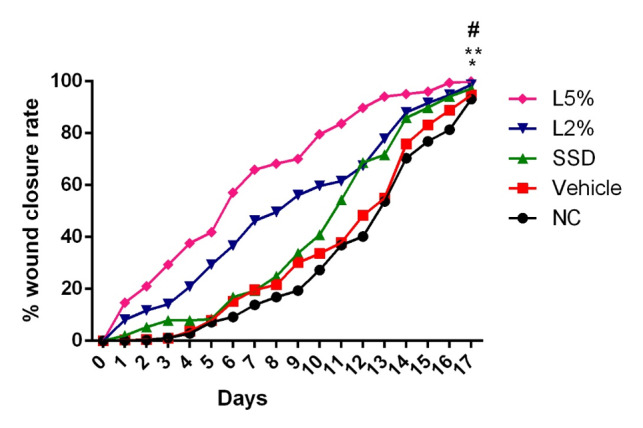
Figure 1. The percent of WCR in rats of all the groups. Findings indicated that luteolin treatment could significantly enhance wound healing compared to negative control (NC) and SSD groups. *P<0.001 vs. NC, **P<0.005 vs. SSD, #P<0.05 vs. L2%

Luteolin Improved the WCR among Rats with Second-degree Burn Wound

The WCR was measured on various days throughout the study, and on the first day, it was considered as zero. On the 17th day (present the complete closer in at least one rat), WCR was significantly higher in rats of the vehicle group than in rats of negative and positive control groups (Figure-1, P<0.01 and P<0.001, respectively). Also, a significant difference in WCR was observed on the final day between the SSD group and both negative control and vehicle groups (P<0.005). Regarding Figure-1, the WCR of luteolin-treated groups demonstrated the greatest wound healing effect compared to the other groups in a dose-dependent manner.

Histopathological Assessment

**Figure-2:**
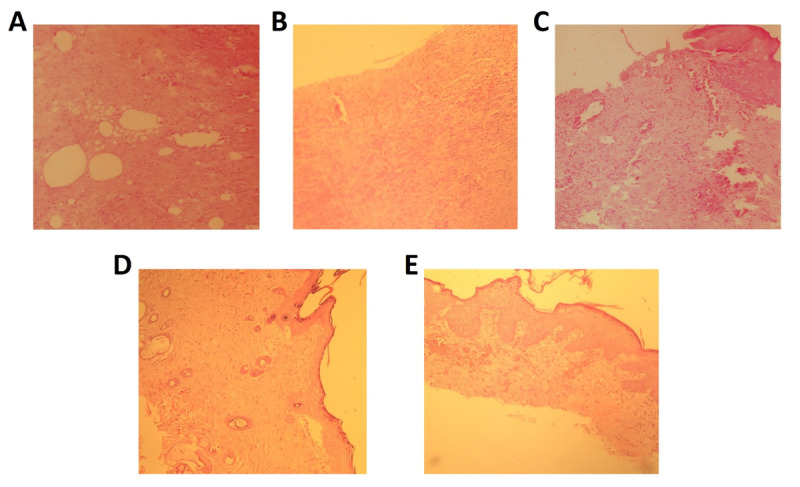
Figure 2. The histopathological findings of rats with second-degree wounds in negative control (A), vehicle (B), SSD (C), and luteolin-treated (D and E) groups (H&E staining, magnification ×200)

In order to measure the severity of tissue damage following burn wounds, histopathology examination, and scoring system were applied. Our findings indicated that the amounts of inflammation and edema in the groups that received luteolin ointments were significantly reduced compared to the vehicle and negative control groups (Figure-2). Indeed, collagen formation and re-epilation were significantly higher in luteolin-treated groups than in other groups. Also, the severity of tissue damage was reduced in rats that received SSD ointment. Indeed, the treatment with luteolin ointments showed a marked increase in the mean score of the L2% (grade2) and L5% (grade3) groups compared with the mean score of the vehicle group (grade1). Also, wound healing improvements in the L5% group were significantly higher than the SSD group (grade2) in a dose-dependent manner (Figure-2).

## Discussion

The results of the current study demonstrate that on the 17th day of treatment, WCR was significantly higher in the luteolin-treated groups compared to both the vehicle and negative control groups as well as the SSD group. This finding suggests that luteolin ointment has a more pronounced effect on promoting wound closure in second-degree burn wounds than standard care alone. Moreover, histopathological examination revealed that rats treated with luteolin ointment exhibited reduced inflammation and edema compared to control groups. Additionally, luteolin-treated groups showed enhanced collagen formation, re-epithelialization, and overall tissue repair, indicating a favorable impact on the healing process of second-degree burn wounds.

In comparison with previous studies [14][15], our findings support the potential benefits of luteolin in wound healing. The significant reduction in inflammation, edema, and tissue damage observed in the luteolin-treated groups are consistent with previous research highlighting the anti-inflammatory and regenerative properties of luteolin [16][17].

In the present study, the inflammation and infiltration of PMN cells in rats treated with luteolin were significantly reduced compared to rats in other groups. In other words, in the L5 group, PMN cells and consequently inflammation were not observed.

The anti-inflammatory biological properties and bioactive compounds derived from the sedab plant, including luteolin and 7-luteolin glucoside were investigated by Caporali et al. [16]. They indicated that among a wide range of flavonoid molecules, luteolin and 7-luteolin glucoside have anti-inflammatory effects in both in-vitro and in-vivo through JAK/STAT3, NF-κB, and other pathways [16].

In the present study, in addition to the anti-inflammatory effects, treatment with luteolin caused a significant increase in angiogenesis and re-epithelialization compared to the control and vehicle groups, as well as rats received standard treatment (SSD). In line with these findings, Zulkefli et al.[18] showed that flavonoids have wound healing properties due to their well-characterized anti-inflammatory, angiogenesis, re-epithelialization, and antioxidant effects.

As previous studies [19][20][21] have shown, the main processes in wound healing are reducing inflammation and improving histopathological damage (including re-epithelialization, angiogenesis, and collagen synthesis at the wound site). It is believed that any factor that able to reduce the time of these processes can accelerate the wound healing process in soft tissues such as the skin [22]. In study by Chen et al. [10], the healing effect of Platycodon grandifloras plant extract, which contains luteolin, was evaluated in comparison with SSD ointment in burn wound healing in an animal model. The results showed that the level of expression of tumor necrosis factor-alpha and interleukin-6 in the serum of rats treated with luteolin and SSD was reduced [10]. On the other hand, the expression of transforming growth factor-beta and vascular endothelial growth factor was higher in the treatment groups compared to the control and burn model rats, which indicates the effectiveness of P. grandiflorus for wound healing [10].

In line with previous studies [10][21][22], the results of our study showed that the collagen deposition as well as re-epithelialization in rats treated with luteolin was significantly increased in a dose-dependent manner compared to other groups, including those receiving SSD.

The observed beneficial effects of luteolin on wound healing may attributed to its diverse pharmacological properties. Indeed, by targeting multiple pathways involved in the wound healing process, luteolin ointment could accelerate tissue regeneration, enhance wound closure, and improve overall healing outcomes in second-degree burn wounds [19][20][21][22]. As the most important limitation, an animal model may not fully demonstrate the complexity of wound healing processes in humans. Hence, further research, including clinical trials is needed to validate the efficacy and safety of luteolin ointment for burn wound management. Additionally, the specific molecular mechanisms underlying the effects of luteolin on wound healing require further investigation to better understand its therapeutic potential in clinical settings.

## Conclusion

Our study demonstrates that luteolin ointment exhibits superior wound healing effects compared to SSD and control treatments in an animal model of second-degree burn wounds. Indeed, the increased WCR, reduced inflammation, enhanced tissue regeneration, and improved histopathological outcomes observed in luteolin-treated groups highlight the potential of luteolin as a promising therapeutic agent for promoting wound healing in burn injuries.

## Conflict of Interest

There are no any conflicts of interest.
